# Ocular manifestations of liver disease: an important diagnostic aid

**DOI:** 10.1007/s10792-024-03103-y

**Published:** 2024-04-15

**Authors:** Riya Patel, Smriti Nair, Hassaam Choudhry, Mustafa Jaffry, Mohammad Dastjerdi

**Affiliations:** Department of Ophthalmology and Visual Science, Rutgers New Jersey Medical School, Newark, USA

**Keywords:** Eye, Hepatitis, Mucopolysaccharidoses, Peroxisomal disorders, Vitamin A

## Abstract

**Purpose:**

This review examined existing literature to determine various ocular manifestations of liver pathologies, with a focus on metabolic deficiencies as well as viral and immune liver conditions.

**Methods:**

Recent data were compiled from PubMed from 2000 to 2020 using keywords that were relevant to the assessed pathologies. Ocular presentations of several liver pathologies were researched and then summarized in a comprehensive form.

**Results:**

Several ocular manifestations of liver disease were related to vitamin A deficiency, as liver disease is associated with impaired vitamin A homeostasis. Alcoholic liver cirrhosis can result in vitamin A deficiency, presenting with Bitot spots, xerosis, and corneal necrosis. Congenital liver diseases such as mucopolysaccharidoses and peroxisomal disorders are also linked with ocular signs. Viral causes of liver disease have associations with conditions like retinal vasculitis, keratoconjunctivitis sicca, retinopathies, Mooren’s ulcer, and Sjogren’s syndrome. Autoimmune hepatitis has been linked to peripheral ulcerative keratitis and uveitis.

**Conclusions:**

Building strong associations between ocular and liver pathology will allow for early detection of such conditions, leading to the early implementation of management strategies. While this review outlines several of the existing connections between hepatic and ophthalmic disease, further research is needed in the area in order to strengthen these associations.

## Introduction

Developing associations between pathologies affecting different organ systems can ultimately help diagnosticians to broaden differential diagnostics. Liver disease, whether congenital or acquired, can result in several manifestations in the eye. By clearly delineating the different ways that liver disease can present in the eye, ophthalmologists and hepatologists can use these associations, allowing for quicker detection and intervention of liver conditions.

Nutritional deficiencies in vitamin A can result in eye pathology, as existing literature has clearly established [1, 2]. However, malabsorptive pathologic states of the liver can also lead to functional vitamin A deficiencies that can be associated with eye pathology. In patients with alcoholic liver cirrhosis, eye symptoms have been reported and have been linked to vitamin A deficiencies [3]. Congenital causes of liver disease, such as biliary defects, peroxisomal disorders, mucopolysaccharidosis, and glycogen storage diseases often present with classic signs of pathologies [4]. Hepatitis C is a viral cause of liver pathology and is also associated with a set of ocular conditions. Autoimmune hepatitis has also been linked to several ocular manifestations, and patients with a history of this condition should receive regular eye exams in order to monitor the development [5, 6].

Hence, given new developments regarding ocular manifestations of liver disease, we seek to summarize the literature surrounding various ocular manifestations of liver pathology in this investigation. The major ocular manifestations of liver diseases thar are discussed are summarized in Table 1. These findings may encourage ophthalmologists to include liver pathologies in their differentials for patients with the associated eye symptoms, as well as encourage hepatologists to have their liver disease patients screened for ocular symptoms.

**Table 1:** Summary of the major ocular manifestations of liver diseases

Disease	Description	Ocular manifestations and structures	References
Vitamin A deficiency	Nutritional deficiets and fat malabsorption decrease vitamin A (specifically chromophore 11-cis-retinal and all-trans-retinoic acid)	Night blindness, retinal dystrophies, photoreceptor cell death, keratomalacia, corneal ulceration, corneal xerosis	[1, 7–11]
Chronic alcohol use/cirrhosis	Chronic alcohol intake decreases oral vitamin A intake and increases vitamin A metabolism leading to vitamin A deficiency	Bitot spots, corneal necrosis, bilateral ocular pain, photophobia, and decreased visual acuity	[12–14]
Galactosemia	Rare carbohydrate hepatic metabolism disease caused by the lack of the galactose-1-phosphate uridylyltransferase enzyme leading to galactose accumulation	Infantile cataracts	[23–25]
GSD type V- McArdle disease	Improper metabolism of glycogen due to PYGM mutations	Pattern dystrophy of retinal pigment epithelium	[26–28]
Biliary Disorders	Variety of cholestatic diseases that affect the bile ducts and gallbladder resulting in reduced bile flow and liver damage	Visual impairment, refractive errors, and optic nerve damage	[31]
Zellweger Syndrome	Fatal inherited defect in peroxisomal biogenesis	Corneal opacification, cataracts, glaucoma, pigmentary retinopathy, and optic atrophy	[41]
Mucopolysaccharidoses	Lysosomal storage disorders result in acumulation of glycosaminoglycans	Amblyopia, strabismus, and large refraction defects, corneal clouding, retinopathy, optic neuropathy, glaucoma, and optic nerve abnormalities	[43, 44]
Niemann-pick disease	Lysosomal storage disorder characterized by sphingomyelinase deficiency	Cherry-red macula, corneal opacifications	[46, 47]
Hepatitis C	Hepatitis C Virus infection due to transmission via blood, sexual contact or occupational exposure	Retinal vasculitis, keratoconjunctivitis sicca, dry eye syndrome, keratitis, scleritis, and retinopathies, Mooren's ulcer, Sjogren’s syndrome	[48–51]
Autoimmune hepatitis	Circulating autoantibodies that attack the liver	Defective tear secretion and stability, dry eye syndrome, peripheral ulcerative keratitis, uveitis	[53–56]

### Metabolic etiologies

#### Vitamin A deficiencies and alcohol use

Although nutritional deficits in vitamin A are uncommon in the United States, liver disease can result in functional vitamin A deficiencies. Vitamin A is a fat-soluble vitamin derived from animal sources such as milk and eggs or from plant sources such as green leafy vegetables and yellow fruits. Ingested retinol is esterified to retinol palmitate, which then travels to the liver [7]. Thus, the liver is closely linked to vitamin A, as this nutrient is stored in hepatic stellate cells.

Vitamin A deficiency results in the deficiency of two metabolites that serve a vital function in the eye: chromophore 11-cis-retinal and all-trans-retinoic acid [1]. 11-cis-retinal, also known as vitamin A aldehyde, plays a key role in the phototransduction pathway, whereas all-trans-retinoic acid is key in tissue development and immune system function [1]. Defects in this pathway can result in a reduction of efficient visual stimulus detection and the accumulation of toxic metabolites in the retina [8]. Deficiencies in vitamin A can therefore result in several manifestations in the eye, including night blindness, retinal dystrophies, photoreceptor cell death, keratomalacia, and corneal ulceration [1]. In addition to these signs, corneal xerosis is also a possible outcome due to vitamin A deficiency caused by liver pathologies [9]. Corneal xerosis is the drying of the cornea and often occurs with acute vitamin A deficiencies [9]. This is attributed to the poor function of glands in the conjunctiva, resulting in the loss of mucus [10]. Another possible outcome is the development of a corneal ulcer or keratomalacia. The spectrum of ocular pathologies that arise from a deficiency of vitamin A is referred to as xerophthalmia [11].

There are several liver pathologies that can result in vitamin A deficiency and can subsequently be associated with the previously mentioned ocular manifestations of the deficiency. In one such example, vitamin A deficiency can result from alcoholic liver cirrhosis. Chronic alcohol intake results in decreased oral retinoid intake while also increasing retinol breakdown by inducing enzyme activity [12]. In patients with cirrhosis, decreased oral intake of vitamin A along with decreased intestinal absorption of vitamin A results in vitamin deficiency that can cause ocular symptoms [13]. Bitot spots are one such manifestation, presenting as keratinous accumulations on the cornea usually preceded by ocular xerosis and ultimately leading to keratoconjunctivitis. If extensive, a vitamin A deficiency in cirrhotic patients can even lead to corneal necrosis [13].

In 2005, a case report by Cruz et al. described a patient with a history of chronic alcoholism that presented with bilateral ocular pain, photophobia, and decreased visual acuity. He was treated for vitamin malabsorption and was given dietary supplements, after which his ocular pathologies significantly improved, and his visual acuity completely recovered [14]. Thus, especially in cases where autoimmune causes of ocular pathologies have been ruled out, vitamin deficiencies should be considered. Other malabsorptive conditions that can result in a vitamin A deficiency include cystic fibrosis, Crohn’s disease, bariatric surgery, short bowel syndrome, celiac disease, and liver disease caused by toxic agents, viruses, and other causes [14].

Moreover, alcohol use disorders can also lead to Wernicke’s encephalopathy which is an acute, neuropsychiatric syndrome characterized by nystagmus, ophthalmoplegia, mental status changes, and difficulties with balance [15]. Wernicke’s encephalopathy is caused by thymine deficiencies which can develop from a combination of factors such as poor diet, decreased gastrointestinal activity, low levels of hepatic storage, and impaired utilization. Its ocular manifestations are nystagmus, an involuntary rhythmic side-to-side, up-and-down, or circular motion of the eyes, and ophthalmoplegia, the paralysis or weakness of the eye muscles [16]. Less common ocular findings with Wernicke’s encephalopathy are optic disc edema, retinal hemorrhage, ptosis, and vision loss [16].

Aside from ocular manifestations associated with alcohol use, vitamin A deficiencies can also occur in chronic cholestatic liver disease, progressive liver diseases that lead to hepatic fibrosis, and liver failure. This group of diseases includes primary biliary cholangitis, primary sclerosing cholangitis, and biliary atresia. The main source of vitamin A for mammals is dietary intake, after which retinoids are stored in the hepatic stellate cells within the liver [17]. Cell injury during the progression of the disease results in the loss of intracellular storage of vitamin A, a mechanism associated with the PNPLA3 gene [18]. The rs738409 single nucleotide polymorphism has been speculated to decrease the activity of this enzyme and has been linked with disease progression of non-alcoholic fatty liver disease [18]. Manifestations of this vitamin deficiency could present in the eye, after which a hepatology consult should be considered to check the health of the patient’s liver.

#### Other metabolic liver diseases

There are a few other liver diseases that result from metabolic deficiencies with ocular features that do not fit into the described categories above and will be discussed in the following section. Wilson’s Disease is characterized by copper buildup in the liver, leading to hepatic failure and liver disease due to the defective metabolism of copper [19]. It involves defective ATP7B protein production and can also be accompanied by cirrhosis and neurological symptoms [20]. This disease can present with ocular signs including the Kayer-Fleisher ring and sunflower cataracts in the lens. The Kayer-Fleisher ring in the eye occurs due to the deposition of copper in Descemet’s membrane and is common in patients with this pathology [21, 22]. With early detection of this disease, early treatment with chelating agents allows for an excellent prognosis.

Another metabolic issue is galactosemia is a rare carbohydrate metabolism disease caused by the lack of the galactose-1-phosphate uridylyltransferase enzyme. Galactosemia presents itself during the neonatal period upon exposure to galactose-containing milk resulting in symptoms such as difficulties feeding, *E. coli* sepsis, hypotonia, renal tubular disease, and elevated liver enzymes [23]. The main ocular symptom that develops in galactosemia is cataracts, which are extremely unusual in infants [24]. A study in 2019 by Rubio-Gozalbo et. al observed cataracts in over 25% of subjects. Of these subjects, approximately half had their cataracts disappear after the removal of galactose from the diet and the other half had residual cataracts even with the change in diet [25]. The presence of cataracts in infants, especially right after feeding, can be indicative of galactosemia as well as increased levels of liver enzymes.

Glycogen storage diseases (GSD), also known as glycogenoses, are congenital metabolic disorders of improper metabolism of glycogen because of enzyme deficiencies, defective transporters, and glycogen degradation. GSDs primarily affect the liver as the liver is the major storage site of glycogen [26]. There are over 20 types of GSD and some present with ocular pathologies. For instance, McArdle disease (GSD type V) is a rare metabolic myopathy due to PYGM gene mutations and may have an association with pattern dystrophy of the retinal pigment epithelium. However, only 3 cases have been reported so further research is required to establish an definite association [27, 28]. Additionally, Allegrini et al. reports a case of a woman with GSD type Ia who presents with ocular changes such as cataract and optic nerve head drusen. However, they indicate that a clear causative mechanism between the two pathologies was not found [29].

### Congenital etiologies

#### Biliary disorders

Biliary disorders refer to diseases affecting the bile ducts, gallbladder, and other structures involved in the production and transport of bile [30]. Many of these diseases can affect multiple organ systems, notably the eye. One study by Fahnehjelm et al. in 2011 looked at the occurrence of ocular issues in children with cholestatic disorders, which are characterized by reduced bile flow and progression of liver damage. The majority of the subjects (68%) experienced ocular manifestations associated with their cholestatic diseases such as visual impairment, refractive errors, and optic nerve damage [31].

More specifically, Alagille syndrome is an inherited multi-organ disease with possible manifestations in the liver, face, eye, heart, and skeletal system. Its hepatic features are characterized by cholestasis, or reduced bile flow, as well as bile ducts paucity, or absence or decreased number of interlobular bile ducts, which can be detected with a liver biopsy within the first year of life [32]. Alagille syndrome also has numerous ocular features such as posterior embryotoxon and optic disk drusen. Posterior embryotoxon is the prominence of the lines of Schwalbe and is found in 56–95% of patients with Alagille syndrome [33]. Although posterior embryotoxon does not have any apparent visual consequences, detecting its presence can be used to diagnose Alagille syndrome. Similarly, optic disk drusen can be used as a clinical diagnostic feature of Alagille syndrome with ocular ultrasound [32]. Other ocular manifestations include angulated retinal vessels and pigmentary retinopathy. More research is required to establish defined relationships between ocular phenotypes and the genetics of Alagille syndrome [34].

Another biliary disease is primary biliary cirrhosis (PBC) is an auto-immune disorder characterized by bile-duct destruction, cholestasis, and even cirrhosis [35]. PBC affects mostly middle-aged women but is often asymptomatic, making diagnosis and early intervention difficult. In symptomatic cases, notable features include fatigue, abdominal pain, and pruritus as well as other medical conditions such as osteoporosis, urinary tract infections, and xanthelasma [36]. Xanthelasma is an ocular manifestation of PBC and is also associated with high levels of cholesterol in the blood. It presents itself with semisolid yellowish deposits along the corners of the upper and lower eyelids. While xanthelasma is benign, many patients can see it as a cosmetic issue and seek medical advice for permanent solutions to remove the patches. Establishing the link between xanthelasma and PBC, especially in middle-aged women, can be a crucial connection to improving the diagnosis of primary biliary cirrhosis.

A common result of biliary abnormalities and gallbladder malfunctions is subsequent hyperbilirubinemia, an excess amount of bilirubin in the blood, and eventually jaundice, a yellowish discoloration of the skin, mucous membranes, and excretions [37]. Hyperbilirubinemia is caused by the improper metabolism of erythrocytes in the liver, which causes bilirubin (the main component of bile) to build up, resulting in jaundice. Moreover, jaundice can also result from the overproduction of bile, the inability of the liver to remove bile pigments from the blood, or the obstruction of the bile ducts [38]. When jaundice presents itself ocularly, it is known as scleral icterus or conjunctival icterus, in which the normally white-colored part of the eye has a yellow tint [39]. Scleral icterus often presents itself prior to other hyperbilirubinemia symptoms and can be the first sign that an underlying biliary condition is present.

Ocular screenings can be extremely helpful in detecting ocular manifestations of underlying biliary disorders, such as posterior embryotoxon or optic disk drusen for Alagille syndrome, xanthelasma for primary biliary cirrhosis, and scleral icterus for hyperbilirubinemia.

#### Peroxisomal disorders

Zellweger syndrome is a peroxisomal biogenesis disorder that is inherited as an autosomal recessive disorder. This pathology is a relatively rare childhood multisystem disorder, presenting in about one in 50,000 newborns, and is associated with several ophthalmic manifestations [40]. As Folz and Trobe describe, manifestations include corneal opacification, cataracts, glaucoma, pigmentary retinopathy, and optic atrophy [41]. This disorder most often causes death within the first year of life.

Rhizomelic chondrodysplasia punctata is another peroxisomal disorder, which presents ocularly with bilateral cataracts. Childhood adrenoleukodystrophy presents with visual deterioration and is inherited through an X-linked pattern. Gradual vision loss presents itself within the first ten years of life due to demyelination of the visual pathway. Primary hyperoxaluria type 1 is associated with parafoveal subretinal pigment proliferation [41]. While many of these conditions present larger, telltale signs of pathology such as seizures and hypotonia, acknowledgment of additional ocular symptoms could assist in developing a differential diagnosis for patients [42]. Furthermore, a thorough understanding of the ocular pathologies that could manifest could result in early detection of these conditions, allowing for early intervention and treatment.

#### Lysosomal disorders

Mucopolysaccharidoses are lysosomal storage disorders that often have ocular symptoms due to the accumulation of glycosaminoglycans [43]. These symptoms are often the first detectable symptoms and are the key to early diagnosis. Common defects include amblyopia, strabismus, and large refraction defects, all of which require early detection in order to offer the best outcome [43]. Other common complications include corneal clouding, retinopathy, optic neuropathy, glaucoma, and optic nerve abnormalities [43, 44]. Corneal clouding is characterized by glycosaminoglycan (GAG) accumulation in the cornea, presenting as yellow-gray granule depositions in the cornea [43]. Deposition of GAG in the anterior segment structures of the eye can increase intraocular pressure which can contribute to glaucoma. Peripheral vascularization is also possible, due to the increased intraocular pressure. This could result in chronic corneal edema, which can produce peripheral vascularization [45]. Retinal dystrophy is also possible, as GAG could accumulate in the retinal epithelium and interphotoreceptor matrix. Further complications also include optic disc swelling, secondary optic atrophy, hyperopia, ocular motility problems, and cerebral visual impairment [45].

Regarding more specific manifestations, Mucopolysaccharidosis Type III, also known as Sanfilippo Syndrome is strongly associated with retinopathy, glaucoma, optic nerve abnormalities, corneal clouding, and late blindness [43]. Mucopolysaccharidosis Type I-H (Hurler syndrome) is strongly associated with corneal clouding, while also being associated with retinopathy, glaucoma, and optic nerve abnormalities to a lesser degree. Mucopolysaccharidosis Type VII (Sly) is associated with colobomas of the iris, and Mucopolysaccharidosis IV (Morquio) is associated with pseudo exophthalmos [43]. These ophthalmic signs of liver pathology could strengthen a diagnosis of a Mucopolysaccharidosis disorder.

Another lysosomal disorder is Niemann-Pick disease which results from acid sphingomyelinase deficiency. The different types of Niemann-Pick disease are categorized on a scale based on the severity of symptoms. Type A or the acute neuronopathic form, is the most common and severe with manifestations such as yellow–brown pigmentation, enlarged lymph nodes, cherry-red macula, and corneal opacifications within the eye [46]. The other categories of Niemann-Pick disease are less severe with less apparent ocular issues. The cherry red spot found in the macula is caused by ballooned, lipid-laden retinal ganglion cells [47]. The cherry red macula is indicative of lysosomal storage disorders and can be narrowed down to Niemann-Pick disease with further screenings.

### Infectious etiologies

#### Hepatitis C

Hepatitis C has a global prevalence and primarily is transmitted parenterally. With the regulation of blood products and blood transmission, the incidence of this disease has been on a decline in the majority of countries [48]. However, there are still other mechanisms of Hepatitis C Virus (HCV) transmission, including occupational exposure and sexual contact [48]. The infection can either be acute or chronic and is treated with direct-acting antivirals that disrupt disease progression [48]. General awareness of ocular manifestations of Hepatitis C infection would facilitate early diagnosis and treatment, serving important implications for clinical ophthalmological practice.

There are strong associations between HCV infection and several eye conditions. In a study by Zeni, Viera, et al., it was found that HCV patients presented with a greater prevalence of lacrimal function abnormalities and higher intraocular pressure than the controls [49]. Eye involvement in HCV can include retinal vasculitis, keratoconjunctivitis sicca, dry eye syndrome, keratitis, scleritis, and retinopathies [48]. The origin of these symptoms could be due to immune responses to HCV antigens and resulting immune complexes. Additionally, the presence of HCV could trigger autoimmune reactions, causing other pathologies [48]. Mooren’s ulcer, or peripheral corneal ulceration, is also associated with Hepatitis C, although this is a controversial association [48, 50]. Mooren’s ulcer is a progressive chronic disorder characterized as idiopathic autoimmune keratitis, affecting the peripheral cornea that can result in severe vision loss [50]. Thus, as HCV progresses, patients should be screened for ocular abnormalities such as ocular surface damage and dry eye, which are more likely to present as hepatic fibrosis progresses [51]. Hepatitis C has also been associated with Sjogren’s syndrome, similar to dry eye syndrome. Sjogren Syndrome is a chronic autoimmune disorder that clinically presents as dryness of the mouth and eye [52].

Thus, patients with HCV infection should be screened for ocular symptoms, and patients with the mentioned ocular symptoms should have HCV infection considered as a part of a diagnostic differential if other risk factors are present.

### Immune etiologies

#### Autoimmune hepatitis

Autoimmune hepatitis is associated with circulating autoantibodies and has been linked to several ophthalmic conditions [53]. In patients with autoimmune liver disease, Citirik et. al found that basal tear secretion and tear film stability were lower and also found symptoms of dry eye among these patients [54]. There is very little existing literature on ocular presentations of autoimmune hepatitis, although there are several case reports suggesting possible ocular associations of autoimmune hepatitis [55]. Eshraghi, Mahtabfar, and Dastjerdi presented a patient case of autoimmune hepatitis with eye irritation that was diagnosed as peripheral ulcerative keratitis [56]. Additionally, Romanelli et. al described an association between autoimmune hepatitis and uveitis [55]. While there is scarce literature on these links, clinicians should be aware of the association in order to aid the early detection of these diseases. However, further research is needed to explore this association and strengthen the link between these pathologies.

## Conclusion

The liver and eye are intimately connected, as there are several ocular signs of liver disease. These ocular changes can be associated with viral, congenital, and autoimmune causes of liver disease and can be important in the early detection of these liver conditions [57, 58]. Aiding early diagnosis could be key to allowing for early intervention and management strategies for these patients, improving patient prognosis and outcome [59]. Many of the described ocular signs are common and could be detected in standard ophthalmic exams. When considered in combination with patient history and present risk factors, ophthalmologists can consult a hepatologist to determine if there is an underlying liver disease. For this purpose, ophthalmologic signs of liver pathology were compiled through a thorough review of the existing literature.

Vitamin A is essential for the health of the eye, and deficiencies of this micronutrient can be seen in malabsorptive states of the liver [1, 2]. In particular, alcoholic liver cirrhosis is often associated with Bitot spots, ocular xerosis, and keratoconjunctivitis [3].

Congenital causes of liver disease can include mucopolysaccharidoses and peroxisomal disorders [4]. Mucopolysaccharidosis is often accompanied by conditions such as amblyopia, strabismus, large refraction defects, retinopathy, glaucoma, and corneal clouding [60]. Peroxisomal disorders such as Zellweger syndrome can present with cataracts, glaucoma, pigmentary retinopathy, and corneal atrophy [61]. While these conditions are often present with larger classic signs of the pathology, strengthening the association between the hepatic and ocular manifestations of these conditions could assist the early implementation of symptomatic treatment [62].

Hepatitis C is another liver pathology with an ocular presentation, including retinal vasculitis, dry eye syndrome, keratitis, scleritis, Sjogren’s syndrome, and keratoconjunctivitis sicca [5, 6]. Furthermore, autoimmune hepatitis has been associated with uveitis and peripheral ulcerative keratitis, although further studies on this topic are needed to strengthen this association [63].

The outlined pathologies demonstrate that the liver and eye are closely related, and associations between their pathologies can aid both ophthalmologists and hepatologists when developing diagnostic, management, and treatment plans [64]. While this paper outlines many of the existing links between hepatic and ophthalmic conditions, further research is needed in order to strengthen these associations and discover new links between these two organ systems.

## Literature search

**Fig. 1:**
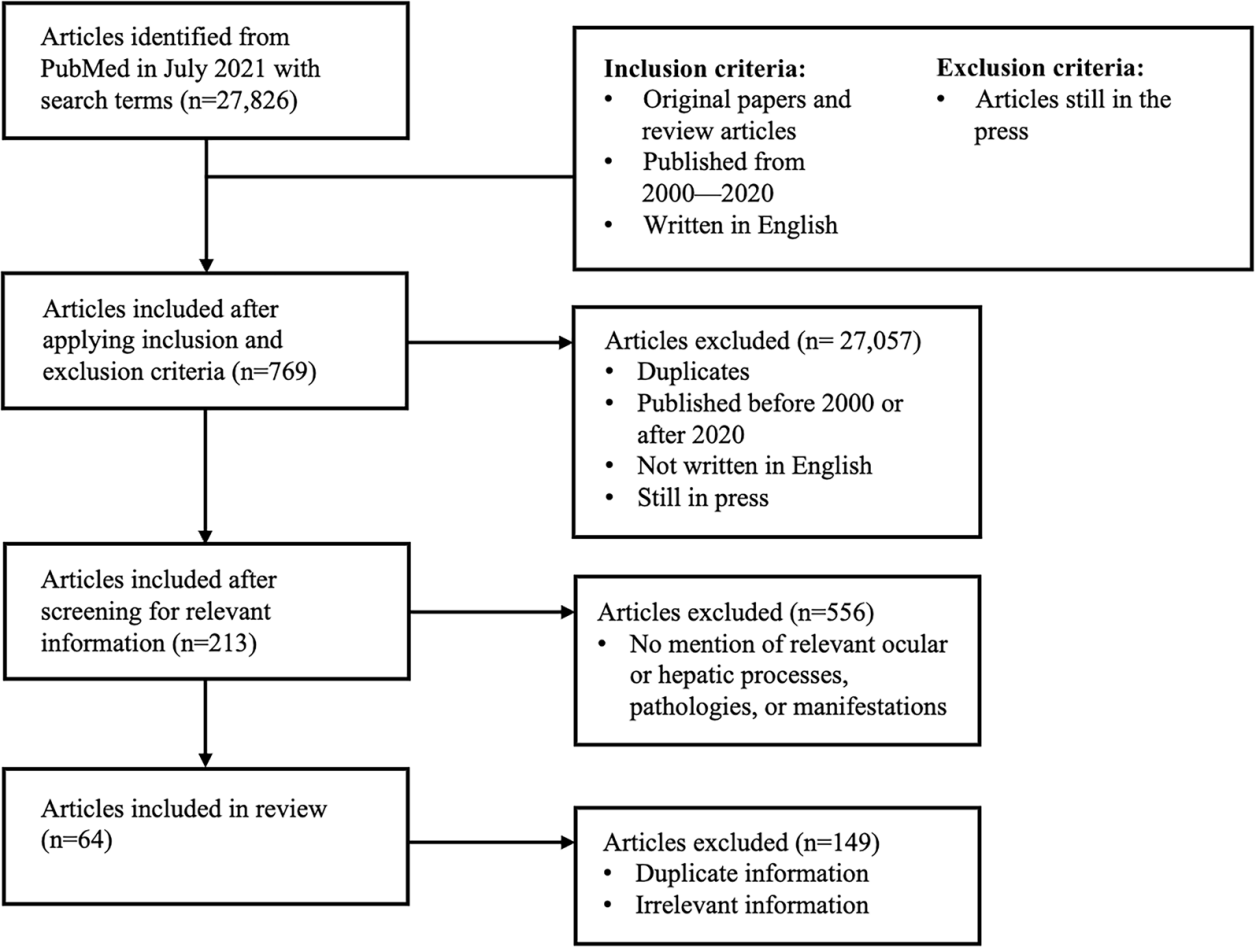
Literature search methodology flowchart considering inclusion and exclusion criteria and the number of articles chosen and rejected from the initial literature search pool

Recent data were compiled from PubMed from 2000–2020 only using keywords that were relevant to the assessed pathologies. After a comprehensive search in July 2021, inclusion criteria were original papers and review articles published from 2000–2020 that contained appropriate information on ocular and liver pathologies. International sources were included. Exclusion criteria were sources that were still in the press, lacked relevancy to ocular and liver pathologies, and outside the time frame of 2000–2020. Some keywords that were searched for include but are not limited to: “ocular manifestations,” “liver disease,” “vitamin A deficiency,” “alcoholic liver cirrhosis,” “chronic cholestatic liver disease,” “Wilson’s disease,” “galactosemia,” “biliary diseases,” “jaundice,” “peroxisomal disorders,” “lysosomal disorders,” “mucopolysaccharidoses,” “McArdle disease,” “hepatitis C,” and “autoimmune hepatitis." A flowchart outlining this methodology as well as the number breakdown of articles included and exluded is shown in Fig. 1. The findings were then tabulated for improved structural organization.
